# Use of theories of human action in recent conservation research

**DOI:** 10.1111/cobi.14461

**Published:** 2025-04-01

**Authors:** Harold N. Eyster, Rachelle K. Gould, Kai M. A. Chan, Terre Satterfield

**Affiliations:** ^1^ Gund Institute for Environment University of Vermont Burlington Vermont USA; ^2^ The Nature Conservancy Boulder Colorado USA; ^3^ Rubinstein School of the Environment University of Vermont Burlington Vermont USA; ^4^ Institute for Resources, Environment and Sustainability University of British Columbia Vancouver British Columbia Canada

**Keywords:** applying theory, behavior, behavior change, conservation social science, human action, human action theory, interdisciplinary theory, social science theory, acciones humanas, aplicación de teorías, cambio conductual, ciencias sociales de la conservación, comportamiento, teoría de acciones humanas, teoría de ciencias sociales, teoría interdisciplinaria

## Abstract

Social sciences are increasingly recognized as useful for reorienting human action toward environmental conservation. Fully realizing the social sciences’ potential requires applying social science methods to conservation challenges and drawing from and building on human action theories from across the social sciences to better understand how and when actions can realize positive social and environmental priorities. We conducted an in‐depth analysis of a bounded, systematically selected set of conservation science peer‐reviewed articles to investigate the prevalence of social science theories of human action in conservation research and whether these theories represent the richness of the social science literature related to human action. We censused papers published in 2023 in *Conservation Biology*, *Conservation Letters*, and *Biological Conservation* and assessed each paper's geographic scope, social science engagement, whether it investigated human action, and weather it explicitly used human action theories and underlying metatheory (i.e., ways of understanding the world and how one gains knowledge of it). Results across 533 papers showed that 32% of papers incorporated social science and that 64% of these social science papers investigated human action. Twenty‐seven percent of these human action papers used explicit human action theories. The theory of planned behavior was the most used explicit theory (17% of action theory papers). The independent self metatheory was the most prevalent; it underlies the theory of planned behavior and focuses on understanding how personal attributes, such as values, shape intentional individual behavior. The prevalence of a few theories and metatheories in these dominant conservation journals may indicate a limited capacity for conservation research to build on previous research, avoid redundant reinvention, and unmask novel applications of social science theory that could reorient human action toward conservation. Human action theory use in conservation might be broadened by changing attitudes on the importance of human action theories for research; incorporating social theory in conservation education; asking reviewers to comment on theory usage and mandating theory reporting; creating spaces for social scientists and theory scholars; providing social scientists and theorists with decision‐making power in organizations; rewarding theory use; recognizing feedback loops among theory use; and replacing colonial and capitalistic approaches to conservation.

## Abstract (es)

Uso de la teoría de acciones humanas en la investigación reciente sobre la conservación

**Resumen:**

Cada vez se reconoce más la utilidad de las ciencias sociales para reorientar la acción humana hacia la conservación del medio ambiente. Para aprovechar plenamente el potencial de las ciencias sociales es necesario aplicar sus métodos a los retos de la conservación; y aprovechar y construir a partir de las teorías de la acción humana de todas las ciencias sociales para comprender mejor cómo y cuándo las acciones pueden materializar prioridades sociales y ambientales positivas. Realizamos un análisis profundo de un conjunto acotado y sistemáticamente seleccionado de artículos revisados por pares de ciencias de la conservación para investigar la prevalencia de las teorías de las ciencias sociales sobre la acción humana en la investigación de la conservación y si estas teorías representan la riqueza de la literatura de las ciencias sociales relacionada con la acción humana. Censamos los artículos publicados en 2023 en *Conservation Biology*, *Conservation Letters y Biological Conservation* y evaluamos el alcance geográfico de cada artículo, su compromiso con las ciencias sociales, si investigaba la acción humana y si utilizaba explícitamente las teorías de la acción humana y la metateoría subyacente (es decir, las formas de entender el mundo y cómo se adquiere conocimiento sobre él). Los resultados de 533 artículos mostraron que el 32% de los artículos incorporaban las ciencias sociales y que el 64% de estos artículos investigaban la acción humana. El 27% de estos trabajos sobre acción humana utilizaron teorías explícitas de la acción humana. La teoría del comportamiento planificado fue la teoría explícita más utilizada (17% de los trabajos). La metateoría del yo independiente fue la más frecuente; subyace a la teoría de la conducta planificada y se centra en comprender cómo los atributos personales, como los valores, dan forma a la conducta individual intencionada. El predominio de unas pocas teorías y metateorías en estas revistas de conservación dominantes puede limitar la capacidad de la investigación en conservación para basarse en investigaciones anteriores, evitar la reinvención redundante y desenmascarar aplicaciones novedosas de la teoría de las ciencias sociales que podrían reorientar la acción humana hacia la conservación. El aumento del uso de la teoría de la acción humana en la conservación podría implicar cambiar las actitudes sobre la importancia de las teorías de la acción humana para la investigación; incorporar la teoría social en la educación para la conservación; pedir a los revisores que hagan comentarios sobre el uso de la teoría y obligar a informar sobre la teoría; crear espacios para los científicos sociales y los estudiosos de la teoría; proporcionar a los científicos sociales y a los teóricos poder de decisión en las organizaciones; recompensar el uso de la teoría; reconocer los circuitos de retroalimentación del uso de la teoría; y erosionar la hegemonía de los enfoques coloniales y capitalistas de la conservación.

## INTRODUCTION

Environmental problems are created, defined, and resolved in large part by people. They are a function of human actions, including behaviors, motivations, practices, collective actions, decisions, and habits (Amel et al., 2017; Dietz et al., 2009; Eyster et al., 2022; Fischer et al., 2012; Mayer, 1982; Watts, 2013). Consequently, the social sciences are increasingly recognized as essential to conservation for their capacity to reorient human action toward effective, equitable, and just pathways (Bennett et al., 2017; Guerrero et al., 2018; Hernandez, 2022; Moon et al., 2018). To this end, journals and scholars increasingly call for engagement with the social sciences (Massarella et al., 2021; Teel et al., 2018).

Social research on human action can contribute to conservation in many ways (Bennett et al., 2017), for example, by explaining how to mitigate hunting bycatch (Blake et al., 2023), how to engage community scientists in the collection of wildlife data (Diekert et al., 2023), or how to demotivate people to own exotic pets (Hausmann et al., 2023). Applications of social science research methods to conservation challenges can be hugely helpful, but they might be even more helpful if they were to draw directly from and build on the many theories across the social sciences that explain what people do, why they do it, and under what enabling or disabling conditions they do it.

For example, in their study of motivations to own exotic pets, Hausmann et al. (2023) leveraged the widely used social science theory of human motivation called self‐determination theory (Ryan & Deci, 2000a). By harnessing this theory, the authors used not just the human motivation data elicited from their study but also decades of research on motivation across many applications to understand people's motivations to own exotic pets. Specifically, the theory may have helped the authors identify the prominence of attachment and other relational dimensions that motivate exotic pet owners.

As this example suggests, theory can aid research in many arguably foundational ways (Bross, 1953; Fawcett, 1978; Littlejohn, 1983). Social science theories of human action (Eyster et al., 2022) may be particularly helpful to conservation because of the complexity of social–ecological systems (such as exotic pet ownership and its constituent problems of exotic pet collection, breeding, ownership, animal health, and zoonosis). Without theory, one would need to study every separate site and issue de novo and would not be able to accumulate a broader understanding of who does what, why, and when (Littlejohn, 1983). There is a place for purely applied and inductive research, but with reference to theory, such research offers the opportunity to contribute to a broader understanding and to ease the burden on future researchers with similar applied questions.

*Theory* has many definitions. In many positivist and quantitative fields, theory most often focuses on the use of quantitative explanatory variables to anticipate outcome variables (reviewed in Sandberg & Alvesson [2020]). But Sandberg and Alvesson (2020) suggest that theory can be more broadly defined and comprehensively organized into 5 different purposes, including explaining (i.e., explain given phenomena), comprehending (i.e., comprehend given but socially defined phenomena), ordering (i.e., categorize indeterminate or ambiguous phenomena), enacting (i.e., produce or reproduce phenomena constructed through process), and provoking (i.e., challenge phenomena constructed and reconstructed through perspectives and vocabularies) (details in Table 1 of Sandberg & Alvesson [2020]). This broader conception of theory enables a more multidisciplinary exploration of theory because the types span positivist to constructivist, quantitative to qualitative, and pragmatic to critical axes. We adopted Sandberg and Alvesson's (2020) definition of *theory* for this review.

Human action theories are a specific class of theories that provide conceptual structures for understanding what drives, changes, creates, or inhibits human action, either as individuals or collectives. Human action theories are diverse, and each has different boundary conditions and relevance (Sandberg & Alvesson, 2020). Prevalent human action theories are often conceptually narrow and pertain only to limited pieces of complex, multiscale systems (Eyster et al., 2022; Naito et al., 2022). Such narrow theories may also rest on dominant and prevailing assumptions about the world, for example, that action is driven by voluntary choices by informed individuals (Shove, 2010). Although these dominant theories may often be relevant, relying too heavily on one set of theories may mask key insights to the human dimensions of conservation problems.

Efforts have been made to make human action theory more accessible, interdisciplinary, and intelligible (Eyster et al., 2022; Gould, Soares, et al., 2023; Stern, 2018), yet researchers have not concomitantly studied the prevalence of these theories in conservation research. There have been no systematic reviews of conservation science engagement with social research, although some reviews suggest that only a small minority of conservation studies address social questions (Evans, 2021; Fazey et al., 2005; Godet & Devictor, 2018; Velasco et al., 2015). Some researchers have examined particular social science fields in conservation and found theory relatively scarce (e.g., in conservation psychology [Wallen & Landon, 2020]). No recent reviews have analyzed engagement with human action theory broadly, though a review from 2005 suggests that in conservation science theory in general (not just social theory or human theory specifically) is scant (Fazey et al., 2005).

To investigate the status of theory, in particular human action theory, in prominent conservation journals, we conducted a systematic literature mapping of articles published in 2023 in 3 conservation journals: *Conservation Biology*, *Conservation Letters*, and *Biological Conservation*. We assessed the overall proportions of papers that incorporated social science and human action. For studies that included these aspects, we further examined how prevalent social science theories of human action were; the types of theories used most widely; and whether the included studies represented a diverse disciplinary and metatheoretical range of social science theories, assumptions, and disciplines (using a recent typology of human action metatheories). We hypothesize that some metatheories and theories are disproportionately represented.

## METHODS

### Sampling

We analyzed papers published in 2023 from *Conservation Biology*, *Conservation Letters*, and *Biological Conservation*. We selected these journals to be consistent with previous reviews of conservation research (Clark & May, 2002; Di Marco et al., 2017; Fazey et al., 2005; Godet & Devictor, 2018; Griffiths & Dos Santos, 2012; Velasco et al., 2015). These journals are all well‐established (published for over 15 years). The former 2 journals have ties to the Society for Conservation Biology (SCB), whose Social Science Working Group (SSWG) was involved in launching the special issue in which this paper appears in celebration of its 20th anniversary. These journals, though their primary focus is not social science per se, are well‐suited for examining the current state of social science theory within the larger conservation field and in reference to the community with which SCB's SSWG has been most engaged.

To enable deep and thorough engagement with this literature, including analyzing the full text, rather than just abstracts or metadata, and to ensure the timeliness of our systematic literature mapping, we censused papers published in 2023. Had we used a search term instead of a census approach, we may have been able to analyze a wider set of journals and articles published over a longer period, but we may have thus artificially limited the scope of what could be identified as social science and would not have had the capacity to engage deeply with the full texts of each social science paper to understand the use (or otherwise) of human action theories.

We used Web of Science to gather peer‐reviewed papers from the 3 journals published in 2023 (accessed 14 March 2024) and the following search terms: “publication date = 2023/2023 AND publication titles = (Conservation Letters OR Conservation Biology OR Biological Conservation), NOT document types = (biographical item OR correction OR book review OR editorial material OR review article).” This search excluded corrections, book reviews, editorial pieces, and review articles. We used Web of Science filters to exclude comments and responses to published articles and letters to the editor. We selected papers published online in 2023 but not necessarily published in a 2023 issue. We chose online publication in 2023 over placement in a 2023 issue because some papers published online in 2023 were not placed in an issue until 2024. Finally, we cross‐referenced this search with journal tables of contents to ensure that we had gathered all relevant papers.

**FIGURE 1:**
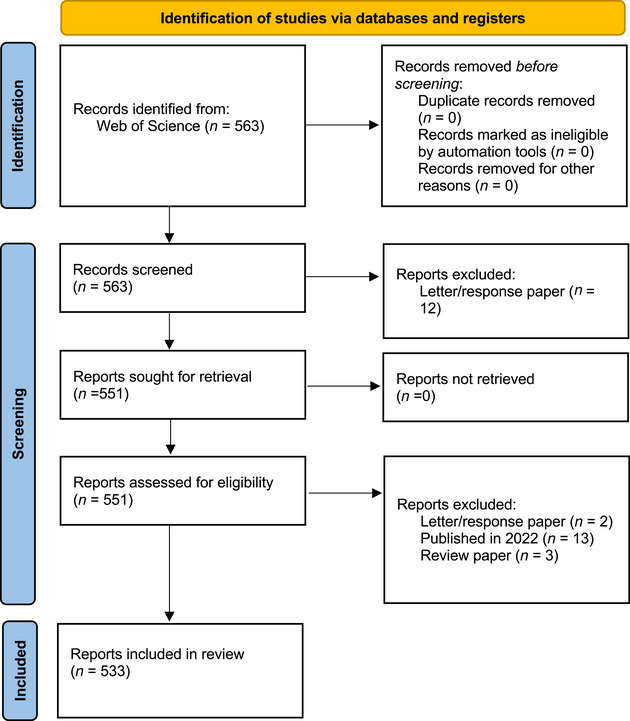
Systematic mapping process, following PRISMA‐2020 (Page et al., 2021), used in a review of social science and human action theories in conservation research.

This search produced 563 papers (Figure 1), including 396, 120, and 47 papers in *Biological Conservation*, *Conservation Biology*, and *Conservation Letters*, respectively.

### Screening

We screened all papers to verify that they met the search criteria. We excluded 30 papers that did not meet our criteria, including 13 papers that were not published online in 2023, 3 papers that were reviews, and 14 papers that were responses to previously published papers, leaving 533 papers for analysis of social science content and theory (Figure 1).

### Analyses

To classify papers as including social science or not, we followed the broad definition of *social science research* established by Bennett et al. (2017), which includes fields ranging from anthropology and ethics to economics and sociology. We excluded papers that included social variables or actors but did not focus on explaining or understanding them. For example, we excluded a study that examined responses of dolphins to tourists swimming with them because it focused on the behavioral responses of the dolphins, not the tourists (Rocha et al., 2023). H.N.E. read titles and abstracts and marked 330 (62%) as not being social science research based on the above criteria and reviewed the full‐text of the remaining papers and marked an additional 33 as not being social science, resulting in a total of 170 social science papers for further analyses (32%).

Second, we took all the papers classified as social science and deductively classified each social science paper's content and human action theory use based on the interdisciplinary synthetic framework developed by Eyster et al. (2022) (Table 1). We chose this framework because it provides a synthetic and metatheoretical typology of human action theories, rather than just behavior theories, which are often more focused on individual, intentional actions and likely to reflect more positivist or quantitative approaches (Shove, 2010). We thus included critical, qualitative, and nonpositivist theories and thereby conceptualized human action broadly: “when individuals, groups, relationships, institutions, societies, etc., undertake (un)conscious decision‐making, (non)volitional behavior, pro/antienvironmental action, motivation, learning, management, pro/antisocial behavior, cooperation, conflict, social movements, societal transitions, cultural norms and practice, habits, compliance, etc.” (Eyster et al., 2022, p. 726). Drawing on previous work, we defined human action theories as a specific class of theories that provide conceptual structures for understanding what drives, changes, creates, or inhibits human action, either as individuals or collectives (Eyster et al., 2022; Sandberg & Alvesson, 2020). There may be social science theories thoughtfully applied to conservation that are not human action theories, but we focused on human action theories.

**TABLE 1:** Human action metatheories (taken from Eyster et al. [2022]) and their uses in conservation research.

Metatheory	Definition	What it enables	Scale of change	Example theories	Use in sample of conservation science papers
Independent self	Metatheory that treats individual behavior as shaped by personal characteristics, such as values, attitudes, traits, beliefs, and worldviews, all of which are treated as independent of and unaffected by external context and structure	Change individual attitudes toward decisions (structure and context are constant)	Small, incremental	Theory of planned behavior (Ajzen, 1985); value–belief–norm theory (Stern, 2000)	Relatively more prevalent among explicit than implicit theories; used to understand values, attitudes, and intra‐individual variations
Independent structure	Metatheory that treats individual behavior as shaped by structures such as culture, institutions, infrastructure, and technologies, all of which are treated as independent of and unaffected by internal processes and personal characteristics	Change institutions to enable change, holding individuals constant	Medium, moderately incremental	Socioecological systems framework (McGinns & Ostrom, 2014); collective action theory in organizations (Bimber et al., 2005)	Rare; used to examine how policies, landscapes, institutions, and other structural factors drive human action
Cognitive needs	Metatheory that assumes the ultimate purpose of human action is survival or evolutionary fitness, which results from the satisfaction of any need associated with the cognitive processing of information	Change cues to harmonize cognitive needs with specific choice (e.g., nudges)	Medium, incremental	Nudge theory (Thaler & Sunstein, 2008); resource‐rational analysis (Lieder & Griffiths, 2020)	Rare; more frequent among papers that lack a geographic focus, reflecting the metatheory's focus on universal cognitive needs
Psychological needs	Metatheory that assumes the ultimate purpose of human action is to produce subjective well‐being, which results from the satisfaction of psychological needs	Intrinsically motivate people, which in turn increases subjective well‐being	Medium, moderately systemic	Self‐determination theory (Ryan & Deci, 2000a); attachment theory (Bowlby, 1969)	Widespread among papers with explicit theories; used to investigate well‐being and motivation; more widespread among papers that lack a geographic focus, reflecting the metatheory's focus on universal psychological needs
Communal needs	Metatheory that assumes social cooperation (e.g., collaboration, collective action, effective governance) can be created by the satisfaction of any number of communal needs	Create adaptive communal, equitable processes	Medium, moderately systemic	Diffusion of innovations (Rogers, 2010); narrative theory (Polletta, 1998)	Prevalent; used to design processes to facilitate group decision‐making and collaboration
Economic needs	Theories share the assumption that the ultimate purpose of action is to maximize utilitarian well‐being (i.e., utility)	Change costs of choices in a static, homogeneous system	Medium, incremental	Rational choice theory (Morgenstern & Neumann, 1944)	Rare; used to understand how people (usually with homogeneous preferences) will react to incentives and to understand market outcomes on behavior
Interdependent	Metatheory that assumes human action is continually created, reinforced, or eroded by an interdependent web of values, identities, positions, habits, goals, needs, experiences, meanings, institutions, cultures, politics, etc.; interdependent web of factors is continually created, reinforced, or erased by human action itself	Identify and intervene in dynamic feedbacks that produce or reproduce social practices	Large, systemic, and adaptive	Extinction of experience (Soga & Gaston, 2016); domestic practice (Hand et al., 2005); environmentality (Agrawal, 2005)	Rare; used to represent feedbacks among action and causes of action
Top down	Metatheory that assumes often hidden, systemic factors unidirectionally shape human action	Identify overarching problem	Large, systemic	Anthroparchy (Cudworth, 2014)	Moderately prevalent; used to engage with the effects of large, often taken‐for‐granted processes, such as neoliberalism, colonialism, materialism, or racism; more common among papers with a global geographic focus, reflecting the metatheory's large‐scale focus

*Note*: A more complete treatment of these theories and metatheories is in Eyster et al. (2022).

We recorded whether a paper was about human action and the names of the human action theories it used. We followed Sutton and Staw (1995) and did not count the following as uses of theory: listing references to existing theory (i.e., naming theories); drawing empirical evidence and data from previous studies to develop hypotheses and objectives (“brute empiricism” [Sutton & Staw, 1995]); including variables or constructs, diagrams, or figures without associated explanation and reasoning; or including hypotheses and predictions.

If the paper included a human action theory, we recorded the metatheory or metatheories (Stillman, 2003) to which the theory belonged, following the metatheory framework developed by Eyster et al. (2022). Each metatheory represents a different set of explanatory drivers, causes, scales, and outcomes of human action. The 8 metatheories (Table 1) are independent self, independent structure, cognitive needs, psychological needs, communal needs, economic needs, interdependent, and top down. We have provided an overview of the metatheories (Table 1) and illustrated how the metatheories are used in conservation science (see “RESULTS” and “DISCUSSION”), but we did not provide a detailed treatment of these theories and metatheories (for such a treatment, see Eyster et al. [2022]).

In addition to these action theory categories, we also classified the geographic focus of each study and wrote a one‐sentence summary. H.N.E. and R.K.G. piloted this deductive approach on a few papers, and then H.N.E. carried it out on the remaining papers.

We are all based in North America and are active researchers in social science and conservation, including human action theories. Our geographic location likely limited our analysis to scholarship borne out of Western and English‐language traditions of conservation (although we intentionally followed the conventions of recent reviews in the field). We acknowledge that conservation can mean many things and that we have excluded much important knowledge and research created in non‐English languages or published in nonglobal‐scope journals (Inzunza et al., 2023).

## RESULTS

**FIGURE 2:**
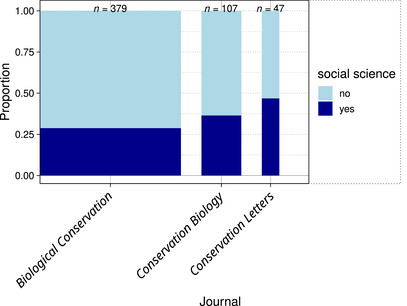
Proportion of papers that were social science in the 3 conservation journals examined in a review of social science and human action theories in conservation research (bar width is proportional to total number of papers in the sample from that journal [Figure 1]).

Nearly one third (*n* = 170; 32%) of papers published in *Conservation Biology*, *Conservation Letters*, or *Biological Conservation* in 2023 conducted social science research (Figure 2). *Biological Conservation* published the most papers and had the lowest ratio of social science papers to nonsocial science papers. *Conservation Letters* published the fewest papers and published the highest ratio of social science papers (Figure 2).

**FIGURE 3:**
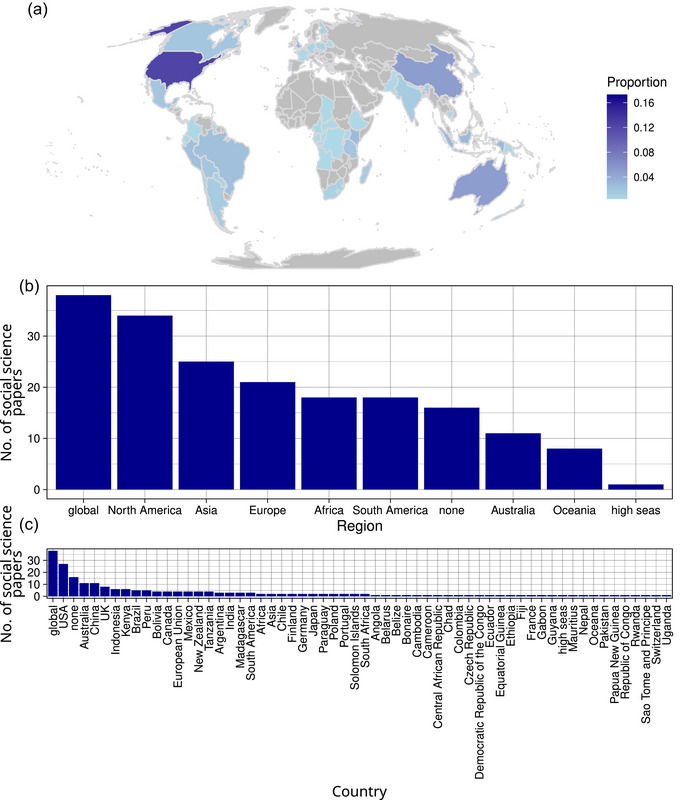
The (a) geographic focus of each conservation social science paper by (b) region and (c) country in a review of social science and human action theories in conservation science research (in [a], continent‐, global‐focused, and place‐agnostic papers are not shown, but are included in the denominator of the proportion calculation).

Fifty‐one countries were studied in our sample of social science papers, with the United States having the most studies (Figure 3b,c). When including broader regions, many papers focused on North America, the entire planet, or had no geographic focus (Figure 3a). Twenty‐three percent of social science papers (23%) were continental or global in scope (Figure 3b).

The majority of social science papers addressed human action (*n* = 108; 64% of papers), while 36% focused on values, justice, or other concepts but did not explain or discuss action. Seventeen percent of social science papers explicitly built on previous human action theories (*n* = 29; 17% of social science papers; 27% of human action papers). Some papers explicitly avoided theory: “Although our dataset was somewhat small and focused on one particular effort, the intent of this study was not to build theory or add to established measures. Rather, we sought to provide a picture of what a conservation‐focused social media effort attracted in terms of audience” (Cavanah et al., 2023, p. 8).

No one theory was widely used. Although our sample of papers was small and only from a single year, the theories that were used multiple times included theory of planned behavior (a behavior theory) (*n* = 5) (Ajzen, 1985), self‐determination theory (a theory of motivation) (*n* = 2) (Ryan & Deci, 2000b), and structured decision‐making (a theory of decision‐making) (*n* = 2) (Gregory, 2012). Authors of 17 human action papers produced their own frameworks but did not connect them to existing theory.

**FIGURE 4:**
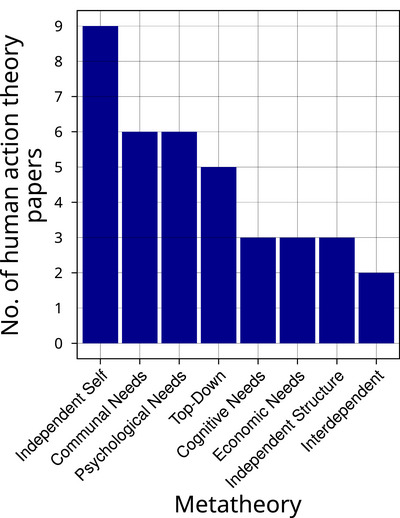
The number of metatheories represented by the explicit human action theories used in conservation social science papers (total number of papers = 29).

**FIGURE 5:**
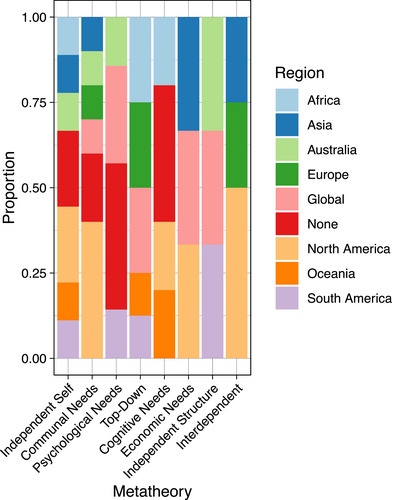
The relative geographic focus of each human action metatheory found in a review of social science and human action theories in conservation research.

The 29 papers in which explicit human action theories were used represented all 8 metatheories developed by Eyster et al. (2022) (Figures 4 & 5; Table 1). Application of these metatheories varied in frequency, but these frequencies should be interpreted with caution given our small sample size. The independent self metatheory was represented in 9 papers, and communal needs and psychological needs were represented in 6 each. Top down was represented in 5 papers; cognitive needs, economic needs, and independent structure in 3 papers each; and interdependent in 2 papers (Figure 4).

### Metatheory overview and usage

Independent self theories, such as the theory of planned behavior, are based on the assumption that individual characteristics drive individual intentional behavior mostly free from outside “context” (Table 1) (Eyster et al., 2022; Shove, 2010).

Independent structure theories assume that infrastructure, landscapes, and social factors shape human action. For example, de Oliveira Caetano et al. (2023) applied an independent structure theory to explain how socioeconomic and country factors determine how many times people search in Google for different biodiversity and conservation terms.

Communal needs theories are based on the assumption that groups have certain needs and that by adopting different principles, practices, and processes, groups can achieve particular types of decisions, collaborations, and collective action (Eyster et al., 2022). Such assumptions appear to be a common pathway for integrating human action into conservation research, at least among the 3 journals we analyzed. For example, Beckman et al. (2023) used a communal needs theory to inform their study of why some conservation easement nonprofits report equity goals, whereas others do not.

Psychological needs theories harness the wealth of information about how psychological needs (such as for belonging) motivate people to act and flourish in environmentally relevant ways. For example, Thompson et al. (2023) used a psychological needs theory to understand motivations and behavior of citizen scientists and how they might be motivated to collect more useful data.

Cognitive needs theories are based on the assumption that cognitive constraints and processes are key to determining human action. For example, Sullivan‐Wiley et al. (2023) applied a cognitive needs theory to determine how to create behavioral interventions across many conservation contexts.

Economic needs theories are based on the assumption that people are rational actors who predictably respond to incentives to maximize their well‐being (i.e., utility, similar to concept of *Homo economicus* [Levine et al., 2015]) (Table 1). This metatheory can show how to intervene in the dominant socioeconomic system to bring about a desired change (e.g., payments for ecosystem services, carbon taxes) or to predict the market outcomes of changes. For example, Thogmartin et al. (2023) used an economic needs theory to estimate how wetland habitat declines might decrease duck hunting and in turn decrease economic activity in duck flyways.

Theories in the top‐down metatheory look beyond the current socioeconomic system to identify the bigger (hegemonic) structures that are often unseen but unidirectionally shape many societies. For example, Ochoa‐Ochoa et al. (2023) used a top‐down theory to understand and challenge inequitable conservation. This metatheory was more prevalent among papers with a global geographic focus.

Theories in the interdependent metatheory enable the integration of feedbacks by assuming that individual feelings and knowledge, structures and technology, and habits and actions themselves all interact interdependently to shape future action (Shove, 2014). For example, Garfinkel et al. (2024) used an interdependent theory to disentangle the positive feedback loops between biodiversity, biodiversity perception, biodiversity conservation, and urban residents. Only 2 papers used this metatheory.

Nearly half (*n* = 13; 42%) of papers that included explicit human action theories built on or critically modified the theories rather than just applying them (i.e., 8% of all social science built on critically modified human action theories). Some modified theory by combining existing theories into integrative theories. For example, Glatthaar et al. (2023) combined the theory of planned behavior and the wildlife tolerance model into an integrative theory.

## DISCUSSION

Human action is crucial to today's conservation challenges and solutions. If conservation researchers and practitioners want to leverage decades of social science research and learn from applied conservation social science research, they must engage with theory. Our census of papers published by 3 leading conservation journals in 2023 showed that such engagement with human action theory is still uncommon (only 27% of papers concerned with human action, which equaled only 5% of our sample's papers overall). We also identified the different human action theories used, as well as the assumptions, or metatheories, that underlie each theory. Although our sample was small, our analyses of these theories and metatheories suggested that theories that focus on individual attributes may be overrepresented in conservation science, whereas those that focus on interdependent feedbacks and nonlinearities may be underrepresented. Our results open doors for potentially new opportunities to apply social science knowledge to conservation challenges.

That 27% of human action papers used human action theory is consistent with previous estimates of theory use in conservation social science, including an estimate of theory usage in conservation psychology (33%) (Wallen & Landon, 2020) and in the *Human Dimensions of Wildlife* journal (32%) (Wallen et al., 2024). Conservation social science generally may be underutilizing theory. However, this usage may be increasing. A 2001 analysis of conservation journals showed that 14% explicitly engaged with theory (including social science and natural science) (Fazey et al., 2005).

This low engagement with human action theories may be partially explained by the cycle of credibility (Hessels et al., 2019; Latour & Woolgar, 1979). This interdependent human action theory posits that scientists use research funds to conduct research that builds their credibility and reputation, then use this increased credibility to do yet more research and further increase their credibility, and so forth. In short, researchers will pay particular attention to investigating and communicating about research that will increase their reputation, and these actions may create a stable cycle of focus on a particular way of doing science. Because conservation science stems largely from ecology (Brussard, 1991; Soulé, 1985) and has only begun to embrace social science (as demonstrated by our result that approximately one third of papers in our sample were social science) (Winkler‐Schor et al., 2024), engagement with social theory may do little to advance conservation scientists’ credibility and thus not be a focus of their work. However, these reward and credibility systems may change as scholars with social science and interdisciplinary reputations are increasingly rewarded by the growing number of interdisciplinary journals, grants, and academic and environmental organization positions. For example, the Nature Conservancy just hired their inaugural Global Director of Human Dimensions Science.

The relative frequency of social research in conservation may be increasing, from a low of 6% on a “social topic” in 2011 (Velasco et al., 2015) up to 32% in our study, though our categorization methods may have differed. This social science growth tracks with broader trends in the field, with increasing recognition of the crucial role that human interactions play in conservation (Bennett et al., 2017; Moon & Blackman, 2014) and that indeed all environmental conditions and conservation are anthropogenic and so require engaging human action (Eyster et al., 2022; McKibben, 1989; Reddy et al., 2016).

Geographic patterns in our 2023 sample are similar to previous studies of conservation more broadly, including the dominance of the United Sates, United Kingdom, Australia, and China (Di Marco et al., 2017). However, Canada and South Africa were less prevalent in our review, whereas Kenya, Indonesia, and Peru were more prevalent. Although our data came from a single year and so may not be indicative more broadly, these patterns likely reflect histories of colonization, wealth, population, and the geographic origins of aspiring global‐scope journals (as aforementioned, all 3 of these journals are published in English and based in North America or Europe) (Das et al., 2013; Ergin & Alkan, 2019; Inzunza et al., 2023). This pattern is present in other fields. For example, a study showed that, all else being equal, economics articles about the United States were more likely than those from other countries to be published in top‐tier journals (like the 3 that we analyzed) (Das et al., 2013).

We identified some papers (*n* = 17) that produced new human action frameworks but did not connect them to extant human action theories. Such novel social science frameworks can be important, especially given the place‐based nature of conservation (Chang, 2016). However, connecting such grounded, applied, or place‐based theories and frameworks to extant theories may be essential for identifying common challenges and solutions. Our results suggest that there may be many opportunities for framework synthesis and theory building in conservation social sciences. Seizing these opportunities may require investing in social science expertise, including by hiring social scientists and including social science and scientists in conservation journals, conferences, and environmental organizations (Moon et al., 2018; Nyssa et al., 2024; Winkler‐Schor et al., 2024). Such theory building and cross‐pollination—that is, theory transferability—could help interdisciplinary fields like conservation science better tackle the crises that they were developed to address.

### Use of metatheories in conservation

The prevalence of the independent self metatheory, and particularly the theory of planned behavior, suggests that not only was engagement with human action theories quite low but also the theories used did not represent the full range of widely applicable social science theories about why people do what they do. The theory of planned behavior was developed in the 1970s and 1980s (Ajzen, 1985; Fishbein & Ajzen, 1975) and has been widely used in psychology and many applied fields, but it has also been critiqued for its low predictive validity and focus on how individual characteristics drive immediate, intentional behavior—behavior that may not always be the most relevant to conservationists (Sniehotta et al., 2014). This is because much conservation‐relevant behavior is not consciously planned; rather, it is the product of existing infrastructure, the decision authority embedded in different institutional designs, or the propelling force of social norms and habits (Colvin et al., 2015; Fielding & Hornsey, 2016; Levine et al., 2015; Shove, 2010). Moreover, the prevalence of this theory also suggests that conservation social science papers may be more likely to adopt quantitative and positivist theories, at least in our sample.

**FIGURE 6:**
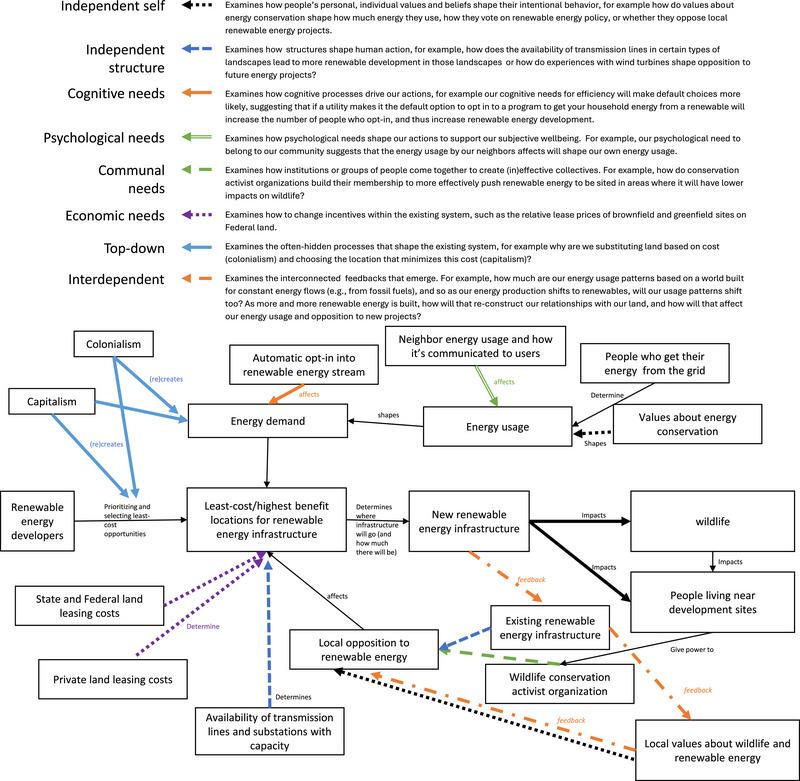
Example of how each human action metatheory applied in reviewed conservation science papers might be used to examine the impacts of renewable energy sites on wildlife and local human communities. Different arrow colors and types of lines differentiate metatheory entry points.

Many have critiqued the overutilization of the independent self metatheory (Schmitt et al., 2019; Shove, 2010). Our results are consistent with this idea that individual attitudes and values receive disproportionate focus given the limited contexts in which these factors determine or explain action. We suggest scholars seeking to engage more explicitly with theory avoid taking up this metatheory as a default and instead adopt theories within a metatheory that is most appropriate for the problem at hand. For guidance on how to select appropriate metatheories, see Table 1 and Figure 6, and Figures 4 and 6 in Eyster et al. (2022).

Our result that cognitive needs and psychological needs metatheories were more common among papers without a geographic focus (Figure 5) was expected because these needs are theorized to be universal and fundamental to every human, rather than varying across different groups and collectives. There may be opportunities for integrating and connecting communal needs with the more fundamental and universal cognitive and psychological needs, for example, by tying a psychological need for belonging with a communal need to feel part of an organization. Doing so may help explain how communal needs translate across different networks of socioecological relationships.

The rarity of the use of the economics needs metatheory may be because rational actor assumptions about people are so dominant in conservation that they are not explicitly stated. This implicitness reflects the long history of ecology–economics collaboration in conservation, as evidenced by widely adopted ideas, such as ecosystem services (Polasky & Segerson, 2009). Although the economic needs metatheory can show how to shift incentives in many current socioeconomic systems, it may not represent marginalized ideas that contravene the dominant economic system nor identify radical reforms of that system (Dempsey, 2016). For example, in applying an ecosystem services framework to examine ungulates in Wyoming (USA), Maher et al. (2023, p. 12), “identified one important and chronically underrepresented group—Tribal members and Native residents—for whom the [ecosystem services and disservices] framework simply fails to capture their understanding of wildlife migrations and their relation to people.” Thus, although economics needs theories may be useful for understanding near‐term behavior and predicting changes in human action that result from small changes in the system, they may fail to navigate more inclusive and transformative pathways for change.

Top‐down metatheories may have been more common among papers with a global geographic focus because of their focus on large‐scale processes, such as colonialism. Yet, many conservation papers seemed to miss opportunities to apply theories from this metatheory, suggesting that it may be underutilized. For example, García‐Roselló et al. (2023, p. 5) examined the geographic distributions of taxonomic information and found that, “[taxonomic information gaps] also reveal the existence of the often observed pattern of geographical bias toward those regions with more taxonomic resources and a long‐standing naturalistic tradition.” Using words such as *bias* rather than connecting these patterns to the many top‐down theories, such as colonialism, capitalism, and imperialism, misses opportunities to investigate and understand the processes that have shaped where “long‐standing naturalistic traditions” are recognized (Inzunza et al., 2023). Employing top‐down theories can improve understanding of the longstanding but often unseen drivers of many of today's conservation challenges (Liboiron, 2021; Moranta et al., 2021; Ruder & Sanniti, 2019).

Researchers have posited that conservation social science research may focus too much on external structures (i.e., the “outer world”) rather than personal feelings and values (i.e., the “inner world”) (Mikołajczak et al., 2023). However, our analysis of human action theories produced different findings. We found that overrepresentation of external structures did not extend to human action theories. Such “inner world” theories may be more prevalent because they are more epistemologically consistent with the dominant positivist approach of conservation science (Roebuck & Phifer, 1990; Shove, 2010), but this may be changing (Moon & Blackman, 2014).

Many have called for greater integration of feedbacks among structures, individuals, and action in environmental social science research (Chan et al., 2020; Eyster et al., 2022; O'Brien, 2018; Shove, 2014). The rarity of the interdependent metatheory suggests that such consideration of feedbacks was rare among our sample. Particularly in the face of rapid socioecological change, engaging with these feedbacks and adaptiveness, as the interdependent metatheory does, may be all the more important.

Our analysis included a number of features that narrow its implications. First, we surveyed only English‐language, “global,” conservation research journals with broad conservation scopes. The journals we selected are not targeted specifically at social science theory and may tend to restrict deeper theoretical explanations as a function of word length restrictions. These journals provide just one idea of what conservation is and what counts as conservation research. Theoretically inclined social science researchers may not believe that these journals’ aims and scope align with their research questions, theoretical approach, reporting standards, or peer‐review expectations. Other environmental journals may thus be better positioned to represent social theory than the ones we chose. For example, had we included other journals, such as *Conservation and Society*, which focuses more on social science, our results may have differed. We suggest that future researchers analyze theory from other journals and compare their results with ours. Nevertheless, the ideas advanced in top‐tier journals are most widely read (and thus shared), and these ideas can become hegemonic, so there is value in tracking the dominant practices in these spaces with the most epistemic power. We believe that our examination of articles in these journals provides a good approximation for how social science theory is used in dominant conservation journals. Yet, we caution readers that our results may not be true of all conservation literatures.

Second, and similarly, the prevalence or dominance of different metatheories in our review does not necessarily represent the prevalence of such ideas among conservationists or land stewards. For example, Indigenous conceptions of human action often focus on relationships and holism (Hernandez, 2022; Salmón, 2000; Watts, 2013), which are more consistent with interdependent and psychological needs metatheories. Yet, these ways of knowing are not privileged in such global journals (for complicated reasons related to ontological and epistemic assumptions, preferences, and power [see below]) and so are likely epistemologically underrepresented among our sample (Weir et al., 2024).

Third, although some theorization occurs implicitly (i.e., scholars approach and conduct their research with a particular set of implicit assumptions about what variables or social conditions are important), these implicit theories are difficult to categorize because they are, well, implicit. Making theories more explicit will improve transparency and facilitate interdisciplinarity and integrative research. It will also enable literature maps, such as ours, to more fully characterize the field's engagement with theory. Fourth, because engagement with human action theories was relatively uncommon, the relative proportions of metatheories in our sample should be interpreted with caution.

### A way forward

Seizing opportunities to leverage human action theories for conservation will require change. This change may be difficult for many reasons. For example, conservation scholars may not know where to start when engaging with human action theory (Figure 6); there may be a lack of theory training in conservation biology courses; supervisors of research students may not be trained in social science methods, methodologies, epistemologies, or theories (Detoeuf et al., 2024; Moon & Blackman, 2014); conservation journal reviewer and editor communities may focus on ecological, rather than social, theory; and social science theorists may be excluded from conservation research organizations and their value may be largely unrecognized (Nyssa et al., 2024; Winkler‐Schor et al., 2024).

Drawing on the 8 metatheories of human action (Table 1), we speculate that efforts to increase human action theory use in conservation could entail first, independent self, changing attitudes toward and raising awareness about the importance of human action theories for conservation research; second, independent structure, incorporating social theory training in conservation biology courses, hiring scholars with this training (Detoeuf et al., 2024; Moon & Blackman, 2014), and increasing accessibility of how to apply human action theories (to help scholars identify and engage with the most appropriate human action theory, we created a schematic [Figure 6] that shows how conservationists can determine which human action theory is most appropriate by thinking about which aspects of the system they want to focus on and how and what types of questions they want to answer in light of the different advantages of the different metatheories [see also Table 1 and Eyster et al., 2022]); third, cognitive needs, creating spaces during the review process for reviewers to easily comment on theory usage and making theory reporting a clear expectation of paper contents; fourth, psychological needs, creating inclusive spaces in conservation conferences, journals, and organizations where social scientists and theory scholars can find belonging, encouraging those who lFead and organize these meetings to recognize the competence and epistemic value of these scholars, and practicing reciprocity between natural and social theorists (Eyster et al., 2024; Kimmerer, 2024); fifth, communal needs, rethinking the ways conservation organizations are run to ensure that social scientists and theorists have decision‐making power and the agency to change those organizations; sixth, economic needs, celebrating and rewarding theory use, for example, by creating awards to recognize theory use in conservation papers, using theory expertise as a criterion in hiring and promotion, and including theory application in conservation grant calls for proposals and evaluation criteria; seventh, interdependent, recognizing the feedback loops among theory use in and among conservation organizations, researchers, and practitioners and ensuring that change efforts acknowledge these connections and tackle them interdependently; and eighth, top down, confronting and seeking to erode the hegemony of colonial and capitalistic approaches to conservation that privilege particular ways of seeing humans in nature and thus certain types of theories (Watts, 2013).

In accordance with the last point, the burgeoning move to better integrate Indigenous ways of knowing offers important promise and challenges with respect to theory. The move toward relational theories of human action is both influenced by and a product of Indigenous knowledge (Eyster et al., 2024; Gould, Martinez, et al., 2023; Tynan, 2021). However, currently the most commonly used theories center around the self (Kimmerer, 2013; Pasternak, 2017). A full treatment of how theory operates in Indigenous knowledge systems is still emerging in the academic literature, but we anticipate that many insights may upend dominant academic knowledge systems. For example, we found that dominant theories of human action are about how *x* drives *y* and so explain individual patterns of behavior. Emerging Indigenous theory may instead reveal systems of inherent rights (Norman, 2019) or natural law (Christie, 2019), which in turn curate or govern behavior in reference to how, when, and what proportion of resources are used and to whom and what future generations responsibility is owed, as well as deeper feedbacks and reciprocity with nature (Kimmerer, 2024; Tynan, 2021). Lastly, what is now called *nature* will likely be theoretically and so empirically reclassified (Jolly et al., 2024) as conservation researchers come to better understand both how fully colonial communities have influenced what conservation scientists have long referred to environmental problems (Chignell et al., 2023).

To foster greater engagement with Indigenous theory, 2 priorities are key—one jurisdictional and the other epistemological. The former requires a repositioning of Indigenous peoples as political–legal entities with territories, laws, and theories of social–ecological well‐being. The latter calls for vastly improved engagement with sovereign Indigenous expert knowledge on its own terms, including through greater understanding about expert knowledge and enduring, orally transmitted observations about social–ecological systems across millennia. These shifts require taking reflexivity much further than currently considered possible or appropriate by most contributors to the knowledge exchange literature. We hope our article can help to open doors and minds to what is considered possible and appropriate and how engagement with broader theory can in turn help broaden, deepen, and strengthen an anticolonial conservation science.
